# Sickle cell related cardiomyopathy and cardiovascular autonomic dysfunction

**DOI:** 10.3389/fcvm.2026.1756623

**Published:** 2026-02-19

**Authors:** Jack Hartnett, Niall Connolly, Sandra Quinn, Ross Murphy, Emma Tuohy, James Curtain, Jens Mogensen, Rose Anne Kenny, Andrew O. Maree

**Affiliations:** 1 Department of Cardiology, St James’s Hospital, Dublin, Ireland; 2 Department of Medicine, Trinity College Dublin, Dublin, Ireland; 3 Department of Cardiovascular Medicine, Mayo Clinic, Phoenix, AZ, United States; 4 Department of Haematology, St James’s Hospital, Dublin, Ireland; 5 Mercer’s Institute for Successful Ageing, St James’s Hospital, Dublin, Ireland

**Keywords:** arrhythima, autonomic dysfunction, cardiomyopathy, sickle cell, sudden death

## Abstract

Sickle cell disease (SCD) is the most common genetic haemoglobinopathy worldwide. Due to advancements in care, SCD patients are living longer. Consequently, there is increased interest in long term sequalae of chronic micro-vascular sickling and resultant end organ damage. Sickle cell cardiomyopathy is an emerging clinical entity characterised by a unique combination of ventricular dilatation, ventricular hypertrophy, diastolic dysfunction and pulmonary hypertension. Additionally, SCD patients have impaired autonomic function which is thought to pre-dispose to vaso-occlusive crises through sympathetic activation and parasympathetic withdrawal during times of physiologic stress. Furthermore, sudden death is a major cause of mortality among patients with SCD, however the mechanism has not been elucidated. This review summarizes the sickle cell cardiomyopathy literature, its relationship with autonomic dysfunction and its association with sudden death.

## Introduction

Normal haemoglobin, known as haemoglobin A (HbA), comprises two *α*-globin and two *β*-globin chains which efficiently carry oxygen in the blood. Sickle Cell Disease (SCD) is an inherited disorder characterised by the dynamic sickling of red blood cells (RBC) secondary to point mutations in the *β*–globin genes which drive abnormal haemoglobin polymerisation to form haemoglobin S (HbS) (Table 1). RBC sickling leads to not only anaemia but also chronic micro-vascular occlusion that results in end organ damage.

**Table 1:** Sickle cell genotypes and phenotypes.

Condition	Genes inherited	Severity	Haemoglobin level (g/dL)	Key clinical features
Sickle Cell Trait (SCT—HbAS)	One normal (HbA) + one sickle (HbS)	Not considered disease	Normal	Usually asymptomatic; occasional painless haematuria; gross haematuria after heavy exercise
Sickle Cell Anaemia (SCA—HbSS)	Two sickle (HbS)	Most common, severe form	6–9	Pain crises, chronic anaemia, microvascular organ damage
Sickle Cell Haemoglobin C (HbSC)	One sickle (HbS) + one HbC	Milder than HbSS	9–11	Mild anaemia, occasional painless haematuria, rare aseptic bone necrosis
Sickle Cell *β*^+^-thalassemia (HbSβ^+^)	One sickle (HbS) + one *β*^+^-thalassemia (some normal β-globin produced)	Milder than HbSS	10–12	Rare pain, mild anaemia, presence of some HbA
Sickle Cell β⁰-thalassemia (HbSβ⁰)	One sickle (HbS) + one β⁰-thalassemia (no normal β-globin produced)	Severe, similar to HbSS	6–9	Similar to HbSS: pain crises, anaemia, organ damage

Approximately 7.74 million people worldwide are living with the disease and global prevalence has increased 41% in the past 20 years (1) because SCD patients are living longer secondary to improvements in care. Over 98% of SCD patients live to adulthood (2). Consequently, long term sequalae of chronic RBC sickling are increasingly recognised including the emerging entity of Sickle Cell related Cardiomyopathy (SCCM). Although most SCD patients live until adulthood, their life expectancy is approximately 22 years less than that of the general population (3). Recent data demonstrate that the main cause of death is end organ damage including heart failure, pulmonary hypertension and sudden cardiac death (4). This review summarizes the SCCM literature, its relationship with autonomic dysfunction and its association with sudden death.

## Methods

A comprehensive literature search was performed in PubMed and Embase in November 2025 using the terms “sickle cell”, “cardiomyopathy”, “autonomic”, “sudden death”. As this is a narrative review, no systematic analysis was performed but results were screened for relevance. Priority was given to publications which focused on pathophysiology and diagnosis. Priority was given to experimental studies and prospective clinical studies over retrospective data, registry studies and case reports. The reference lists of included studies were also screened for relevant publications.

## Pathophysiology of sickle cell cardiomyopathy

**Figure 1:**
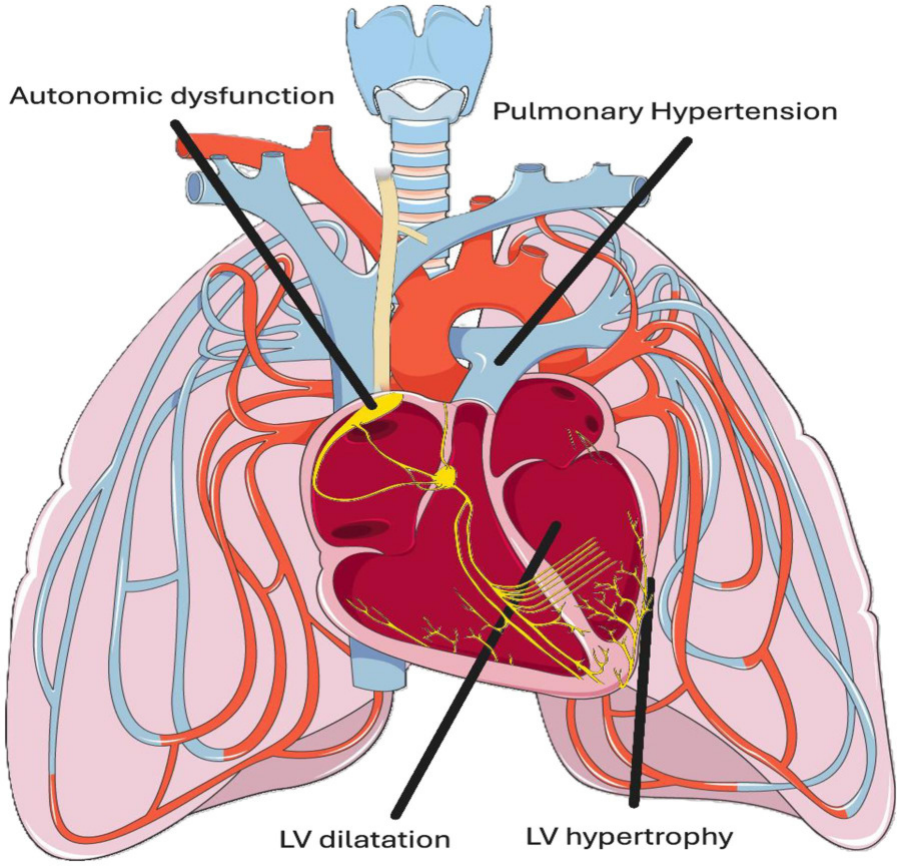
Schematic of sickle cell related cardiomyopathy (SCCM) and associated cardiovascular autonomic dysfunction. SCCM is derived from a unique combination of left ventricular (LV) hypertrophy, LV dilatation, diastolic dysfunction and pulmonary hypertension (PH). Cardiovascular autonomic dysfunction in the form of altered heart rate variability (HRV) and increased systemic vascular resistance (SVR) during vaso-occlusive crises is well established. Adapted from “Pulmonary circulation” by Servier Medical Art (https://smart.servier.com/), licensed under CC BY 4.0.

SCCM is a unique cardiomyopathy characterised by left ventricular dilatation (LVD), left ventricular hypertrophy (LVH), diastolic dysfunction and pulmonary hypertension (PH) (5) (Figure 1).

Numerous studies have identified LVH and LVD on both echocardiography and cardiac magnetic resonance (CMR) imaging (6, 7). Prolonged exposure to chronic anaemia drives a high output cardiac state, which results in eccentric myocardial remodelling that leads to both hypertrophy and dilatation. The degree of cardiac remodelling correlates well with severity of anaemia (6). Longstanding systemic hypertension (thought to be secondary to high cardiac output and primary systemic arterial microvascular disease) also contributes to LVH and LVD. In one study the mean left ventricular end diastolic volume index (LVEDVi) was 124 mL/cm^2^ in SCCM patients (with a mean LV mass of 77.2 g/cm^2^) vs. 78.7 mL/cm^2^ (with a mean LV mass of 51.6 g/cm^2^) in healthy controls (7). Of note, relative wall thickness (RWT) is not commented upon in this study.

Diastolic dysfunction in SCCM is secondary to diffuse myocardial fibrosis. In one imaging study of children with SCD, average extracellular volume of the myocardium (a surrogate measure of myocardial fibrosis), as measured by T1 mapping on CMR, was significantly increased in 100% of SCD patients compared to age and sex matched controls (8). Furthermore, there was a significant positive association between high extracellular volume on CMR and diastolic dysfunction on echocardiography. Both high extracellular volume and diastolic dysfunction were associated with increased frequency of vaso-occlusive crisis.

Studies have investigated the aetiology of the diffuse fibrosis evident in SCCM. CMR studies have shown that myocardial iron overload in SCCM is uncommon (8–10). In one study that comprised 38 patients with SCD, only 1 patient had evidence of myocardial iron overload on CMR (7). SCD patients had a 21% lower myocardial perfusion reserve index compared to healthy controls—implicating microvascular dysfunction as a potential contributor. In a smaller study of 5 SCD patients, CMR identified abnormal perfusion reserves and diffuse myocardial fibrosis in all patients (11). None had evidence of iron overload. It is believed that the repeated episodes of vaso-occlusive crises drive microvascular dysfunction and myocardial fibrosis through the release of damaging reactive oxygen species (ROS) and free radicals. In experimental mouse models of SCD, histopathological analysis demonstrates progressive myocyte loss and myocardial fibrosis (12). At the molecular level there is up-regulation of genes involved in oxidative stress and hypoxia. Together this suggests that the progressive cardiac dilatation and diastolic dysfunction seen in SCCM is driven in response to chronic oxidative stress and chronic hypoxia.

PH is a common sequela of SCD. Right heart catheterisation studies estimate the prevalence of in SCD at 8%–55% (13–15). When classified haemodynamically, 52.6% of SCD patients with PH have pre-capillary PH and 47.4% have post-capillary PH as per a recent review of multiple studies (16). Of note, the prevalence of combined pre-capillary and post-capillary PH was not commented upon. Overall, this suggests that SCD related PH is driven through multiple mechanisms. The high cardiac output state of SCD raises pulmonary pressures via increased flow through the pulmonary vasculature (17). Additionally, pulmonary venous pressures are persistently raised as a result of chronic LV pressure overload from diastolic dysfunction (18). Indeed, left atrial enlargement (LAE) secondary to LV fibrosis and restrictive physiology is a common finding in SCCM (19). LAE can drive left atrial hypertension which in turn causes pulmonary venous hypertension. Similar to myocardial fibrosis, generation of free radicals and ROS during vaso-occlusive crises drives primary pulmonary arterial vasculopathy (20, 21). Micro-thrombi due to sickling can cause chronic thromboembolic pulmonary hypertension (CTEPH) in the pulmonary arterial tree and pulmonary veno-occlusive disease (PVOD) (22, 23). Histopathological analysis of SCD lung tissue has identified features of pulmonary arterial hypertension (PAH), CTEPH and PVOD in the same specimen (24).

Although similar to related cardiomyopathies such as high output cardiac failure or iron overload cardiomyopathy, SCCM is unique in its combination of LVD, LVH, diastolic dysfunction and PH. Ultimately, at the clinical level patients with SCCM commonly present with heart failure. Given that systolic dysfunction in SCCM is rare while diastolic dysfunction is common, heart failure with preserved ejection fracture (HFpEF) is more common than heart failure with reduced ejection fraction (HFrEF). Although all four cardiac chambers can be enlarged in SCCM, contractility of the ventricles is usually preserved (25). PH and diastolic dysfunction are independent predictors of mortality in SCD. In one study in which 141 SCD patients underwent tissue doppler echocardiography, diastolic dysfunction was associated with mortality with a risk ratio of 3.5 (18). When both pulmonary hypertension and diastolic dysfunction were present, the risk ratio for death increased to 12.0 (18).

Professional societies recognise the emerging entity of SCCM and have provided best practice guidelines to clinicians on who to investigate for SCCM and PH (26). The American Society of Hematology (ASH) advises against an echocardiogram in asymptomatic patients but does acknowledge this recommendation is based on low certainty evidence (26). ASH recommends an echocardiogram in SCD patients with symptoms and signs of heart failure or PH. Furthermore, right heart catheterisation should only be considered in patients with a tricuspid regurgitant jet velocity (TRV) ≥ 2.5 meters/second (m/s) on echocardiogram along with an abnormal N-terminal pro-B natriuretic peptide (NT-proBNP) or abnormal six-minute walk distance (6MWD). If NT-proBNP and 6MWD are within reference ranges, serial echocardiograms should be performed until TRV ≥ 2.9 m/s, at which point right heart catheterisation is recommended regardless of 6MWD and NT-proBNP values.

## Sudden death

Autopsy studies have identified sudden death as a leading cause of mortality in SCD and determined that it is often preceded by cardiopulmonary complications (4, 27). In one study premorbid conditions included acute chest syndrome in 58.1%, PH in 41.9%, heart failure in 25.6%, myocardial infarction in 20.9% and arrhythmia in 14% of patients (4).

Focusing specifically on arrhythmia, there is a clear theoretical basis for arrhythmogenesis in SCCM. Myocardial fibrosis is an established substrate for ventricular tachyarrhythmia. When combined with hypoxaemia, oxidative stress, acidosis and pain during an acute sickle cell crisis, the conditions for arrhythmogenesis and consequently sudden cardiac death are generated.

In one study of 100 SCD patients who underwent a 24-hour Holter monitor, ventricular arrhythmia (defined as sustained or non-sustained ventricular tachycardia, >500 premature ventricular contractions in 24 h or a history of ablation for ventricular tachycardia) occurred in 22% of patients (28). Global longitudinal strain (GLS) detected on echocardiography was independently associated with ventricular arrhythmogenesis which the authors propose reflects the development of pro-arrhythmic myocardial fibrosis. Multiple CMR and histology studies, across a broad range of cardiac conditions, have demonstrated that GLS on echocardiography is a sensitive marker of myocardial dysfunction and is associated with myocardial fibrosis (29–32). Preclinical studies also support this concept. In an experimental mouse model of SCCM, the QT interval prolongs as cardiomyopathy progresses—thus augmenting ventricular arrhythmia risk (33).

Chronic inflammation is well established in SCD. Chronic inflammation of the myocardium in SCCM, particularly mediated by interleukin-18 (IL-18), is felt to drive altered expression of cell membrane ion channels and thus alters ventricular myocyte automaticity (33). Of note, in a mouse model, ventricular arrhythmia predominated from the right ventricle (RV). The authors hypothesised that SCD mediated PH and PH mediated increased RV afterload may be the driving factors as myocardial fibrosis patterns did not differ substantially between RV and LV (33).

Continuous cardiac monitoring indicates that up to 80% of patients experience an arrhythmia during a sickle cell crisis (34). Among this cohort, 67% experienced ventricular arrhythmia of which 7% developed ventricular tachycardia (34). It makes sense that arrhythmia and sudden death occur most commonly during acute crises when there is haemolysis and reactive oxygen species production that may provoke electrical instability in an already fibrosed myocardium.

Multiple publications have demonstrated a link between arrhythmias in SCCM and mortality (35, 36). In one study of over 800,000 SCD hospitalisations (data from the National Inpatient Sample in the United States), SCD patients who experienced an arrhythmia had a 2.53X increased risk of all-cause mortality (35). In a more recent database study from the same National Inpatient Sample, the development of arrhythmia in hospitalised SCD patients was associated with markedly increased risk of all-cause mortality (odds ratio 53.6) (36). Although these studies are registry derived and thus have clear limitations such as potential coding error, the strong positive association between arrhythmia and all-cause mortality in SCD patients is highly suggestive of an underlying mechanistic link.

## Pathophysiology of autonomic dysfunction

The autonomic nervous system (ANS) is the unconscious component of the nervous system which regulates key physiologic processes such as heart rate and peripheral vascular tone (37). The ANS is dysregulated in many diverse disease processes (38).

Early theories of SCD pathophysiology proposed that prolonged transit time through the microvasculature is essential to the development of vaso-occlusive crises (39). The longer the transit time, the greater the opportunity for sickling and vaso-occlusion. The ANS is known to modulate peripheral vascular tone, particularly that of arterioles, and may play an important role in SCD and vaso-occlusion in particular.

Although primarily derived from observational or highly protocolized experimental data, there is growing evidence for ANS dysfunction in SCD. Early studies demonstrate marked parasympathetic withdrawal and increased sympathetic tone during vaso-occlusive crises (40, 41). Pain, distress and anxiety induce sympathetic activation and can trigger vaso-occlusion (42). The ensuing vaso-occlusive crisis drives more pain which further promotes sympathetic activation and microvascular vasoconstriction, thereby creating a positive feedback loop that amplifies pain, distress and anxiety. Thus, parasympathetic withdrawal and sympathetic hyper-activation offer a mechanistic link between ANS dysregulation and SCD. ANS dysfunction may explain why some patients experience more frequent and more severe vaso-occlusive crises compared to others.

SCD is associated with ANS hyper-reactivity. In one study, deep breathing and sighing induced dramatic vasoconstriction in SCD patients but not in controls (41). In another, breath holding induced marked vasoconstriction (43). In a third, application of thermal pain stimuli to one arm induced a global sympathetic response which was more profound in SCD patients than in controls (44). In a follow-up study, SCD patients had faster vasoconstriction compared to controls and slower recovery between episodes of thermal pain stimulation (45). The degree of ANS hyper-reactivity in SCD has been associated with disease severity and clinical outcomes (46).

The head up tilt (HUT) test is a well validated research tool to assess autonomic function. During the test, rapid orthostatic changes induce initial hypotension as blood pools in the abdomen and lower limbs. This is followed by an increase in heart rate (parasympathetic withdrawal) and peripheral vascular resistance (increased sympathetic drive) to maintain blood pressure and cerebral perfusion. In one study, SCD patients, non-SCD anaemic patients and control patients underwent HUT testing (47). Physiological responses were characterized in four categories: (1) dual cardiac and peripheral response (2) cardiac predominant response (3) peripheral predominant response (4) subthreshold cardiac and peripheral response, based on their change in heart rate and blood pressure. Interestingly, SCD patients produced a peripheral predominant response. The authors hypothesise that SCD patients who tend to produce a peripheral predominant response (i.e., vasoconstrict in response to orthostatic stress) are at higher risk of vaso-occlusive crises secondary to their vasoconstrictive tendencies (47).

Heart rate variability (HRV) is another commonly utilised research metric to assess ANS function. Although the peripheral vasoconstrictive response is heightened among SCD patients compared to healthy controls, cardiac autonomic function appears to be reduced (48, 49). Numerous publications have demonstrated reduced HRV among SCD patients vs. controls—particularly during vaso-occlusive crises (48, 49). This is in keeping with the prior HUT study which identified lower baseline cardiac parasympathetic function among SCD patients (47). The resting heart rate is increased and the ability to dynamically alter chronotropy is reduced in response to physiologic stress in SCD patients.

The aetiology of ANS dysfunction in SCD is unclear but most likely reflects microvascular ischemia which drives autonomic neuropathy. One study found an association between blood viscosity and degree of HRV impairment in SCD patients (50). Although no causation is demonstrated, this result suggests that increased viscosity in the microvasculature may cause neuronal hypoxia which ultimately impairs ANS function. Further work is required to build on this preliminary study.

## Future directions

The cardiomyopathy of SCD is unique—characterised by LVD, LVH, diastolic dysfunction and PH. Multi-modal imaging studies have demonstrated the association between this structurally unique cardiomyopathy and patient outcomes. However, the complex haemodynamics of SCCM remain unexamined. Future studies, using advanced imaging techniques such as quantitative first pass perfusion CMR or positron emission topography (PET), are needed to quantify the haemodynamics of SCCM and uncover its effect on clinical outcomes.

Although both SCCM and ANS dysfunction are well characterized in SCD, the likely interplay between them and their potential combined role in sudden death in this vulnerable patient group has not been established. Many years ago the increased risk of arrhythmia during a vaso-occlusive crisis was confirmed (34). As described above, pain during a vaso-occlusive crisis drives sympathetic activation and microvascular vasoconstriction (44, 51). Resultant ischaemia leads to inflammatory mediator release. Recent evidence from murine models implicates interleukin (IL)-18 as a mediator of ventricular arrhythmia in SCCM. This occurs through prolongation of the action potential and QT interval (33). Retrospective data shows that a prolonged QT interval and ventricular arrhythmias are significant predictors of mortality in SCD cohorts (52). Similarly, thrombocytopenia (which often occurs during a vaso-occlusive crisis due to platelet consumption) is also associated with ventricular arrhythmogenesis in SCD (28). Based on the above preliminary evidence alone, there is a clear rational for further study of the relationship between ANS dysfunction, SCCM and sudden death in SCD. Of particular interest would be whether SCD therapies, which have been proven to reduce vaso-occlusive crisis frequency, have any effect on slowing or reversing SCCM and ANS dysfunction development.

## Conclusions

Sudden death is common among SCD patients. However, its mechanism remains unclear. This review parses emerging data surrounding SCCM, ANS dysfunction and their potential combined mechanistic role in sudden death. Further research is required to establish the relationship with the ultimate goal being prevention of sudden death aamong SCD patients.
